# CYP24A1 Deficiency Presenting With Hypercalcemia and Hypervitaminosis D in Pregnancy: A Case Report

**DOI:** 10.7759/cureus.100639

**Published:** 2026-01-02

**Authors:** Susanne U Trost, Lynn Burmeister

**Affiliations:** 1 Endocrinology, Diabetes and Metabolism, University of Minnesota Medical School, Minneapolis, USA

**Keywords:** 1'25-dihydroxyvitamin d, 24-hydroxylase deficiency, cyp24a1 deficiency, cyp24a1 gene mutations, non-parathyroid hypercalcemia, pregnancy and hypercalcemia, vitamin d, vitamin d intoxication, vitamin d supplementation, vitamin d toxicity

## Abstract

The increasing use of vitamin D supplementation may have unintended consequences for individuals who exhibit heightened sensitivity, leading to supraphysiologic serum vitamin D levels. This is a 32-year-old woman with non-parathyroid hormone-dependent hypercalcemia during pregnancy. Laboratory evaluation showed high 25-dihydroxyvitamin D levels, indicating vitamin D intoxication. Very high calcitriol levels, with a high 25-hydroxyvitamin D:24,25-dihydroxyvitamin D ratio, indicated CYP24A1 deficiency. The patient did not have any history of hypercalcemia, including during prior pregnancies. A homozygous variant of CYP24A1 (c.1490A>C; p.His497Pro) was found. Pregnancy increases the risk of hypercalcemia in individuals with a CYP24A1 mutation by a physiologic increase of 1-α-hydroxylase. Vitamin D and calcium supplementation can be an additional trigger for hypercalcemia in these patients, as seen in our patient. Vitamin D deficiency during prior pregnancies might have been protective from hypercalcemia.

This report raises awareness of a subgroup of women at risk for adverse events from vitamin D and calcium supplementation during pregnancy. Checking calcium levels currently is not routine during pregnancy; however, it should be considered more often for symptomatic women.

## Introduction

Use of vitamin D supplements is common and is generally viewed as safe [1,2]. However, sensitivity to vitamin D supplementation has long been recognized, including the infrequent occurrence of hypercalcemia in infants in the 1950s in Britain [3], during a time of high vitamin D supplementation and fortification. Changes in vitamin D metabolism were found by Schlingmann et al. in 2011, describing inactivating mutations in the 1,25-dihydroxyvitamin D (1,25(OH)₂D)-metabolizing enzyme CYP24A1 in a cohort of infants with idiopathic infantile hypercalcemia (IIH), as well as in otherwise apparently healthy infants with hypercalcemia triggered by vitamin D prophylaxis [4].

CYP24A1 deficiency (Infantile Hypercalcemia Type 1) was recognized as a rare yet important etiology of non-parathyroid hormone (PTHi)-related hypercalcemia [5], being associated with low or low-normal PTHi and elevated 1,25(OH)₂D. In addition to CYP24A1 deficiency, other etiologies of hypercalcemia with increased 1,25(OH)₂D and low PTHi include (1) sarcoidosis and other granulomatous diseases, (2) elevated PTH-related protein, (3) vitamin D intoxication [6], (4) SLC34A1 defects in renal phosphorus channels and hypophosphatemia [7], and (5) Williams-Beuren syndrome, with developmental delay, characteristic facial features, and increased risk of hypercalcemia, the pathomechanism of which is not clearly understood [8].

CYP24A1 deficiency is associated with low 24,25-dihydroxyvitamin D (24,25(OH)₂D), the degradation product of 25-hydroxyvitamin D (25(OH)D). The elevated ratio of 25(OH)D to 24,25(OH)₂D above 80 has been found to differentiate hypercalcemia related to CYP24A1 deficiency from vitamin D intoxication [9,10]. Variability in the CYP24A1-associated clinical phenotype has been reported, including time of onset of hypercalcemia and the presence of nephrocalcinosis and nephrolithiasis. Diet, sun exposure, infancy, pregnancy, and vitamin D supplementation were found to influence IIH manifestations [7]. It has been acknowledged that IIH has become a misnomer for patients with pathologic variants of CYP24A1, who frequently present later in life [11,12]. 

Pregnancy is associated with several physiologic changes in calcium metabolism, including doubling or tripling of 1,25(OH)₂D concentration [11], due to upregulation and placental expression of 1-α-hydroxylase and PTHrP expression in the breast and placenta [13]. Patients with CYP24A1 deficiency are found to have worse clinical and biochemical features during pregnancy than age-matched pregnant peers [5]. In a previously reported cohort, 13 women with 29 pregnancies and pathologic mutations in the CYP24A1 gene, accompanied by hypercalcemia, presented with mental status changes, pancreatitis, seizures, hypertension, nephrolithiasis, worsening renal function, rhythm disturbances, and obstetric complications, including preeclampsia and eclampsia [11]. 

Neonatal complications have been reported, including preterm birth, intrauterine growth restriction, renal calcifications, and fetal demise. Nevertheless, most infants born to mothers with pregnancy-associated hypercalcemia due to CYP24A1 mutations do not develop IIH, as the condition is typically inherited in an autosomal recessive manner. In some newborns, transient hypercalcemia has been observed, generally resolving within days to a few weeks. Episodes of transient hypocalcemia have also been described, and isolated reports have noted transient neonatal hypoglycemia. Measurement of ionized calcium every other day for one to two weeks in the newborn, as well as glucose checks, was recommended [11].

With the advent of widespread vitamin D supplementation for its multiple proclaimed health benefits, hypercalcemia due to exogenous vitamin D intoxication has become a concern. Sustained high-dose vitamin D treatment of 100,000 units per day was associated with hypercalcemia in 11%-20% of patients [6]. However, hypercalcemia has also been reported with moderate vitamin D supplementation, at 3,200-4,000 units daily, in a review of larger studies [14]. 

We present a case of a pregnant woman with hypercalcemia during pregnancy, vitamin D intoxication from moderate vitamin D supplementation, and an elevated 25-(OH)D to 24,25-(OH)₂D ratio.

## Case presentation

**Figure 1:**
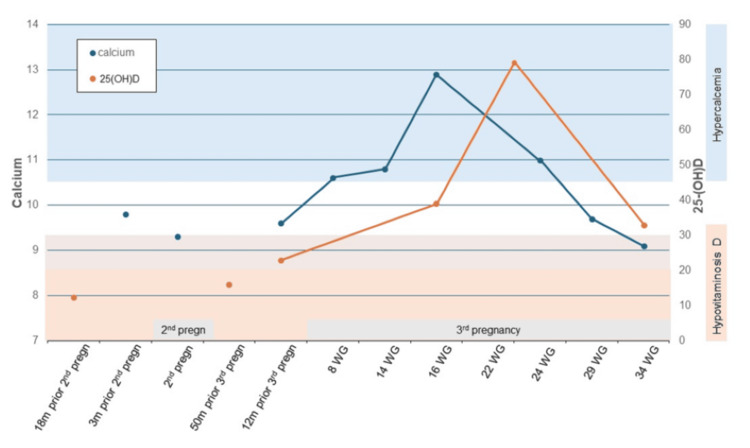
Calcium in Relation to Vitamin D Levels and Pregnancy Status Reference: calcium, 8.8-10.4 mg/dL; 25(OH)D, 30-52 ng/mL. Pregn: pregnancy; WG: weeks’ gestation; 25-(OH)D: 25-hydroxyvitamin D

A 33-year-old woman presented with hypercalcemia, first noted during the first trimester of her third pregnancy. Calcium was normal during her previous pregnancy 10 years prior, as well as prior to conception of the third pregnancy. The first elevated calcium level, 10.8 mg/dL (8.8-10.4 mg/dL), was noted at 10 weeks of gestation. Her pregnancy was complicated by hyperemesis gravidarum in the first trimester. The calcium peaked at 12.9 mg/dL in the second trimester, at 16 weeks of gestation (Figure 1). The patient was advised to stop prenatal vitamins, as well as calcium carbonate, which she had been taking for heartburn. In the second trimester, at 24 weeks, the patient was admitted for preterm labor, experiencing vertigo, nausea, and vomiting. Calcium at the time was 11.0 mg/dL, albumin 2.9 g/dL (reference 3.5-5.2 g/dL), with calculated calcium of 12.8 mg/dL. A C-section was performed at 38 weeks and 0 days. Treatment of hypercalcemia included avoidance of vitamin D and calcium supplements and intravenous hydration.

Evaluation of hypercalcemia in the second trimester revealed very high 1,25-(OH)₂D, >200 pg/mL (reference 19.9-79.3 pg/mL), with undetectable PTHi and parathyroid-related peptide, and normal phosphorus (Table 1). 25-(OH)D₃ was 81 ng/mL, 25-(OH)D₂ <5 ng/mL, total 25-(OH)D 86 ng/mL (reference 19.9-52 ng/mL). An elevated 25-(OH)D:24,25-(OH)₂D ratio of 98.55 (reference <80), based on Mayo Reference Laboratory measurements of 24,25-(OH)₂D 0.69 ng/mL, 25-(OH)D₃ 68 ng/mL, and 25-(OH)D₂ <4 ng/mL, indicated CYP24A1 deficiency (Table 1). Genetic testing, with sequencing and deletion/duplication analysis of the CYP24A1 gene by Invitae Lab (Invitae, San Francisco, CA, USA), revealed a homozygous variant (c.1490A>C; p.His497Pro).

**Table 1:** Calcium and Vitamin D Metabolites Related to Pregnancy Status Reference: Calcium, 8.8-10.4 mg/dL; 25-(OH)D total, 30-52 ng/mL; 1,25-(OH)₂D, 19.9-79.3 pg/mL; 25-(OH)D:24,25-(OH)₂D ratio, <80. *Processed at Mayo Reference Laboratory: 25-(OH)D, 24,25-(OH)₂D, 25-(OH)D/24,25-(OH)₂D ratio. 25-(OH)D: 25-hydroxyvitamin D; 1,25-(OH)₂D: 1,25-dihydroxyvitamin D; 24,25-(OH)₂D: 24,25-dihydroxyvitamin D; Pregn: pregnancy; WG: weeks’ gestation; H: high; I: insufficient; L: low

	18m prior 2nd preg	2nd preg	50m prior 3rd pregn	12m prior 3rd pregn	8 WG	14 WG	16 WG	22 WG	24 WG	29 WG	34 WG
Calcium	-	9.8	-	9.6	10.6H	10.8H	12.9H	-	11.0H	9.7	9.1
25-(OH)D	12.3L	-	16L	23I	-	-	39	86H/68H*	-	-	33
1,25-(OH)_2_D	-	-	-	-	-	-	>200H	>200H	-	>200H	187H
24,25-(OH)_2_D	-	-	-	-	-	-	-	0.69*	-	-	-
25-OHD:24,25-(OH)_2_D	-	-	-	-	-	-	-	98H*	-	-	-

The patient did not have a history of kidney stones or nephrocalcinosis. There is no family history of hypercalcemia; therefore, additional testing could not be pursued. Outside of this third pregnancy, the patient has documented hypovitaminosis D, including around the time of her second pregnancy.

## Discussion

This is a 33-year-old woman with hypercalcemia during her third pregnancy, associated with vitamin D intoxication, lower-than-expected 24,25-(OH)₂D, and a high 25-(OH)D:24,25-(OH)₂D ratio, suggesting a possible CYP24A1 deletion or mutation [10,15]. Hypervitaminosis D usually does not lead to elevated 1,25-(OH)₂D levels [6,16]. Reported exceptions occurred with erroneously very high vitamin D doses, a multitude of times the upper recommended dose [17,18]. In a cohort of 72 patients evaluated for hypercalcemia with low PTH, 35% were found to have CYP24A1 variants [15]. In these patients, a 25-(OH)D:24,25-(OH)₂D ratio exceeding 80 - consistent with our patient’s results - was indicative of biallelic CYP24A1 mutations. Individuals with this defect exhibit heightened sensitivity to vitamin D because impaired 24-hydroxylase activity limits the breakdown of both 25-(OH)D and 1,25-(OH)₂D. As a result, even moderate vitamin D intake can precipitate toxicity. The changes in vitamin D metabolism during pregnancy further increase the risk of hypercalcemia in these individuals. The absence of hypercalcemia during our patient's previous pregnancies likely relates to prior vitamin D deficiency.

Avoidance of vitamin D supplementation, sun exposure, calcium supplements, and calcium-rich foods is considered the cornerstone of chronic treatment of CYP24A1 deficiency. Multiple medical treatment options have been reported; however, they lack randomized or case-control studies. Medical treatment can utilize the modulatory effects of azoles (ketoconazole, fluconazole, or itraconazole) as modulators of Cyp27B1 activity, or rifampin as a Cyp3A4 inducer, as well as bisphosphonates, calcitonin, or cinacalcet [5]. 

In our patient, genetic testing revealed a CYP24A1 c.1490A>C mutation, reported as a variant of uncertain significance (VUS). The CYP24A1 c.1490A>C variant has also been reported in one other patient who presented with hypercalcemia in pregnancy. This patient was heterozygous, with the second mutation being an intronic variant, c.544-17G>A [19]. These two reports support CYP24A1 c.1490A>C as a pathogenic variant exhibiting a recessive phenotype. Computational simulations found the CYP24A1 c.1490A>C variant to have a high probability of being damaging according to PolyPhen analysis, but to be tolerated according to SIFT (Sorting Intolerant From Tolerant) analysis, presenting conflicting evidence about its potential impact on protein function [20].

Screening for family members of patients with CYP24A1 deficiency carrying a VUS mutation should be done with a cautious, multi-pronged approach, combining genetics and clinical data. Clinical screening includes serum calcium, PTHi, and urine calcium-to-creatinine ratio. However, heterozygote carriers might not be identified with clinical screening alone.

Multiple clinical findings in our patient suggest a pathogenic CYP24A1 variant that impairs normal metabolism of 25-(OH)D and 1,25-(OH)₂D. She exhibited persistently high 1,25-(OH)₂D, low 24,25-(OH)₂D, and a markedly increased 25-(OH)D:24,25-(OH)₂D ratio (98.55), all measured during a period of hypervitaminosis D. Although stopping vitamin D supplementation gradually lowered her 25-(OH)D concentration, her 1,25-(OH)₂D level remained high, further supporting a CYP24A1 mutation [6].

## Conclusions

This case illustrates how vitamin D supplementation can affect a patient with an underlying defect in vitamin D metabolism. Women with vitamin D metabolic defects may be without baseline hypercalcemia or nephrocalcinosis, and only present during pregnancy with hypercalcemia and associated complications. Differences in lifestyle, including vitamin D and calcium intake, can lead to different presentations during pregnancies in these women.

Checking calcium levels currently is not routine during pregnancy; however, it should be considered more often for symptomatic women. Measurement of the 25-(OH)D:24,25-(OH)₂D ratio is an essential tool to identify 24-hydroxylase deficiency in patients with non-parathyroid-related hypercalcemia without granulomatous disease or malignancies.
